# Predominant constitutive CFTR conductance in small airways

**DOI:** 10.1186/1465-9921-6-7

**Published:** 2005-01-17

**Authors:** Xiaofei Wang, Christian Lytle, Paul M Quinton

**Affiliations:** 1 Dept. Pediatrics, Medical School, University of California, San Diego, San Diego, CA USA; 2 Dept. Biomedical Sciences, University of California, Riverside, CA USA

## Abstract

**Background:**

The pathological hallmarks of chronic obstructive pulmonary disease (COPD) are inflammation of the small airways (bronchiolitis) and destruction of lung parenchyma (emphysema). These forms of disease arise from chronic prolonged infections, which are usually never present in the normal lung. Despite the fact that primary hygiene and defense of the airways presumably requires a well controlled fluid environment on the surface of the bronchiolar airway, very little is known of the fluid and electrolyte transport properties of airways of less than a few mm diameter.

**Methods:**

We introduce a novel approach to examine some of these properties in a preparation of minimally traumatized porcine bronchioles of about 1 mm diameter by microperfusing the intact bronchiole.

**Results:**

In bilateral isotonic NaCl Ringer solutions, the spontaneous transepithelial potential (TEP; lumen to bath) of the bronchiole was small (mean ± sem: -3 ± 1 mV; n = 25), but when gluconate replaced luminal Cl^-^, the bionic Cl^- ^diffusion potentials (-58 ± 3 mV; n = 25) were as large as -90 mV. TEP diffusion potentials from 2:1 NaCl dilution showed that epithelial Cl^- ^permeability was at least 5 times greater than Na^+ ^permeability. The anion selectivity sequence was similar to that of CFTR. The bionic TEP became more electronegative with stimulation by luminal forskolin (5 μM)+IBMX (100 μM), ATP (100 μM), or adenosine (100 μM), but not by ionomycin. The TEP was partially inhibited by NPPB (100 μM), GlyH-101* (5–50 μM), and CFTR_Inh_-172* (5 μM). RT-PCR gave identifying products for CFTR, α-, β-, and γ-ENaC and NKCC1. Antibodies to CFTR localized specifically to the epithelial cells lining the lumen of the small airways.

**Conclusion:**

These results indicate that the small airway of the pig is characterized by a constitutively active Cl^- ^conductance that is most likely due to CFTR.

## Background

Most, if not all, forms of chronic obstruction pulmonary disease (COPD) as well as asthma begin in the small airways. While the pathogenesis of small airway diseases is poorly understood [1,2], it is generally accepted that the fluid and electrolyte transport properties of the epithelia lining these peripheral bronchioles play a crucial role in maintaining normal airway hygiene and patency. Some argue that these fluids are the primary defense because coupled with the ciliated escalator they form the first mechanism for clearing the airway of foreign debris and noxious agents.

At the same time, almost nothing is known with certainty about the transport properties of distal airway epithelia or how fluid movements help maintain hygiene. No doubt, the paucity of understanding is due to the inaccessibility and the fragility of the tissue. Most concepts of the mechanisms and functions at this level have been taken from findings in the upper respiratory tract or from the larger cartilaginous ringed structures of the trachea and bronchi [3-7]. More extrapolations have been made from primary cultures of the same sources [8,9]. Two previously published attempts were made to measure electrolyte transport parameters in isolated segments of small airways dissected from the peripheral airways of sheep [10-12] and pigs [13,14]. However, in these studies the electrical signals, reflecting underlying transport properties may have been severely muted by tissue trauma during dissection and preparation. For standard electrophysiological studies of epithelia, dissection of the bronchiole would seem mandatory in order to maintain control of solutions on both sides of the epithelium. In order to minimize trauma, however, we attempted to microperfuse small bronchioles (i.d. 0.5–0.8 mm) in the periphery of pig lung without dissection. Unfortunately, since the bronchioles are embedded in a parenchyma of bronchioli and alveoli, this approach sacrifices control of the contra-luminal solution. Nonetheless, under this condition, we now find striking improvements in electrophysiological responses and strong evidence of a highly Cl^- ^selective conductance that dominates the electroconductive properties of this epithelium, that is most probably duo to CFTR.

## Methods

### Tissue

Lungs were excised intact immediately after sacrifice of young pigs (30–60 kg). Lungs were maintained inflated through a ligated plastic tube connected to an aquarium air pump (~1 L/min) to maintain a positive airway pressure of 10–14 cm-H_2_O. The assembly was wrapped in a plastic bag and transported from the abattoir to the laboratory (<60 min) in an insulated box chilled with ice. In the laboratory, small pieces of about 0.5 cm^3 ^were cut from the peripheral lung parenchyma, usually from along the costal diaphragmatic ridge of the lower lobes. In general, the freshest tissue gave the best responses although some tissue responded well after 6–8 hours of storage in a cooled environment.

### Microperfusion

**Figure 1:**
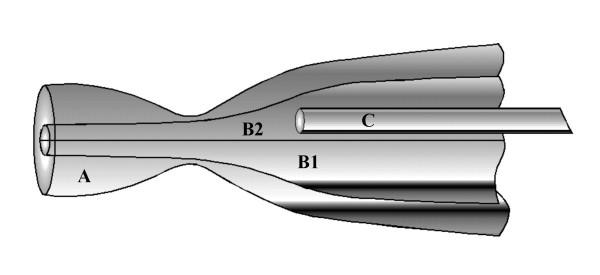
Pipette assembly for microperfusing segments of undissected bronchiole. The bronchiole is held in outer large pipette (A) by suction. An inner, septated cannulating pipette provides current passing capacity through one barrel (B_1_) and perfusing fluid to the duct lumen through the opposite barrel (B_2_), which also contains a small cannula pipette (C) that allows changes of perfusing solutions.

Under a dissecting microscope, the opening to a small airway was visualized on the proximal cut surface of a small block of lung tissue. The airway opening was then cannulated with a system of two concentric micropipettes [15-17] with tips fabricated so that the identified open end of the bronchiole could be aspirated into the outer pipette. (Fig. 1). Simultaneously, a double barreled inner pipette was inserted into the lumen of the bronchiole for delivering experimental solutions and monitoring electrical potentials through one barrel while constant current pulses were delivered through the other barrel [18].

### Solutions

The perfused airways were *intact *and therefore remained embedded in the mass of connective tissue and air filled alveoli that normally surround the bronchi *in vivo*. The surrounding parenchymal tissue effectively prevented changing the solution in contact with the serosal surfaces of the airway epithelium, which *in vivo *is the extracellular fluid and in the* intact* preparation could not be readily removed. NaCl Ringer solution is designed to mimic mammalian extracellular fluid. Therefore, we used NaCl Ringer in the bath to establish electrical continuity with the serosal surface of the bronchiolar epithelium during the entire experimental period. The Ringer solution contained in (mM): Na^+ ^(~155), K^+ ^(4.5), Mg^2+^(1.2), Ca^2+ ^(1.0), PO_4_^3- ^(3.5), Cl^- ^(152), SO_4_^2- ^(1.2), Glucose (5) buffered to pH 7.4 with NaOH. For ion diffusion studies, 150 mM of Cl^- ^was replaced with an equivalent amount of gluconate (taken as impermeant), HCO_3_^-^, NO_3_^-^, I^-^, or Br^-^. Luminal solutions perfusing the bronchiolar airway were rapidly changed as needed via a manifold distributing stores of the above solutions through a needle tube to the tip of the perfusing pipette (Fig. 1). Agonists were added to solutions (in μM) as needed as forskolin (1), IBMX (100), ionomycin (1), ATP (100), and adenosine (100). Inhibitors were added (in μM) as needed as amiloride (10), NPPB (100), CFTR_Inh_-172 (5) [19,20], GlyH-101 (50) [21] (CFTR_Inh_-172 and GlyH-101 were generous gifts from Dr. A. Verkman, University of California, San Francisco, CA.).

### Electrical Measurements

The basic electrical circuit for recording potentials and conductance during microperfusion has been described previously [18]. The lumen of the bronchiole can be considered as a conductive core of fluid (perfusate) surrounded by an insulating epithelium.Unfortunately, the complex arborizing geometry of the bronchiole make it impossible to calculate the specific conductance of the epithelium from cable analysis as is possible with straight, unbranching tubes like sweat ducts and renal tubules. Thus, in the present protocol, the current pulse induced voltage deflections reflect the total resistance of the preparation and can only be used to compare changes in the epithelial resistance within the same preparation when identical solutions are present in the lumen and bath; e.g., pre- and post-drug application.

Although we recorded the total resistance (R_t_) of the system, which includes the summed resistances of the epithelium (including parallel shunts through it) plus the core resistance of the lumen plus the extracellular fluid resistance, the resistance of the epithelium relative to R_t _was small (even after floating the current passing circuit), and therefore changes in the epithelial resistance were obscured. Consequently, the response of TEP's was taken as the primary indication of the permeability properties of the epithelium.

### Temperature

The bathing solution was maintained at 35 ± 2°C.

### mRNA expression

Total RNA was isolated from the dissected bronchioles of 4 pigs by using RNeasy Mini Kit (QIAGEN Inc. CA). RNA was reversely transcribed using Sensiscript RT Kit (QIAGEN Inc. CA). The resulting first-strand cDNA was directly used for PCR amplification (TaqPCR Core Kit, QIAGEN Inc. CA). The conditions for PCR reactions were as follows: 3 min at 94°C (initial melt); 35 cycles of 1 min at 94°C, 1 min at 55–60°C, 1 min at 72°C and then 72°C 10 min (final extension). For the negative control, RT-PCR was performed in the absence of RT. The PCR products were analyzed by agarose gel electrophoresis stained with ethidium bromide.

The primers were constructed on the basis of the published cDNA sequence of CFTR, ENaC, NKCC1 and β-Actin from GenBank. Since the pig gene sequence was not complete, primers were obtained from the human accordant gene, in which highly conserved regions were selected. The pairs of primers for CFTR (accession no. NM_000492) were sense 5'-TCCTAAGCCATGGCCACAA-3' and antisense 5'-GCATTCCAGCATTGCTTCTA-3'; sense 5'-GCCTGGCACCATTAAAGAAA-3' and antisense 5'-CTTGCTCGTTGACCTCCACT-3', which generated a 197-bp and 171-bp CFTR PCR product respectively; for α-ENaC (Z92978) were sense 5'-CAACAACACCACCATCCAC-3' and antisense 5'-TAGGGATTGAGGGTGCAGA-3', which generated a 225-bp PCR product; for β-ENaC (NM_000336) were sense 5'-TGCTGTGCCTCATCGAGTTTG-3' and antisense 5'-TGCAGACGCAGGGAGTCATAGTTG-3', which generated a 277-bp PCR product; for γ-ENaC (X87160) were sense 5'-TCAAGAAGAATCTGCCCGTGA-3' and antisense 5'-GGAAGTGGACTTTGATGGAAACTG-3', which generated a 237-bp PCR product; for NKCC1 (U30246) were sense 5'-TCCAGGTAATGAGTATGGTGTCAG-3' and antisense, 5'-GTTAAGATGTAGCCACGAAGAGGT-3', which generated a 205-bp PCR product; and for β-Actin (BC004251) were sense, 5'-TTCAACTCCATCATGAAGAAGTGTGACGTG-3' and antisense, 5'-CTAAGTCATAGTCCGCCTAGAAGCATT-3', which generated a 312-bp PCR product. All primers showed products closely corresponding to the predicted size for expression of RNA transcripts for these genes.

### Immunocytochemistry

The bronchioles were dissected and then fixed in ice-cold 4% formaldehyde buffered in phosphate at 4°C for 3 hours, infiltrated with cryoprotectant (30% sucrose in PBS) overnight, and frozen in OTC medium (Triangle Biomedical Sciences) at -35°C. Sections of 5 μm thickness were cut on a cryostat microtome (Thermo Electron) and mounted on glass slides (Fisher Superfrost Plus). Antigen retrieval was performed using a pressure cooker (10 min in 10 mM citrate buffer, pH 6). To reduce autofluorescence, sections were treated for 20 min with 1.5% sodium borohydride in PBS. Sections were incubated sequentially with blocking solution (30 min), primary antibody (overnight at 4°C), and secondary antibodies conjugated to Alexa Fluor-488 and/or -546 (Molecular Probes). Confocal images were acquired with a Zeiss LSM-510 microscope and assembled using Adobe Photoshop. CFTR was labeled with rabbit antibody R3194 (courtesy of C. Marino), and ENaC with a rabbit antibody against the β-subunit of human ENaC (kindly courtesy of C. Fuller and D. Benos). Tight junctions were labeled with a mouse antibody against the junction-associated protein zonula occludens-1 (Zymed). Nuclei were stained with TO-PRO-3 (Molecular Probes).

### Statistical treatment

Differences in mean measurement were assayed by applying the Student T test to paired or unpaired means as appropriate. A probability (P value) of ≤ 0.05 was taken as significantly different.

## Results

### Basic electrical properties

**Figure 2:**
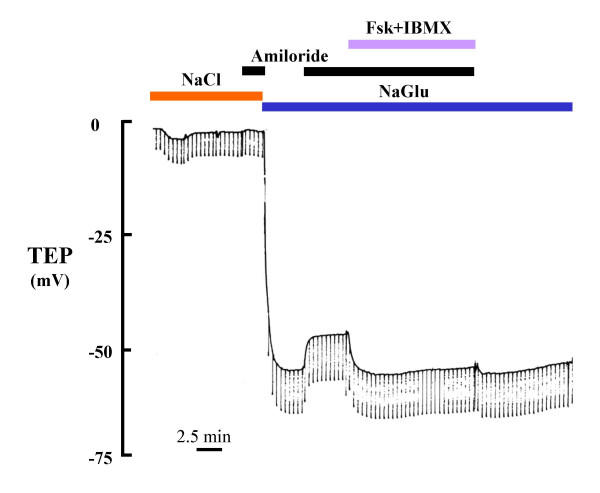
Effect of amiloride and Forskolin (Fsk, 5 μM) + IBMX (100 μM) on transepithelial potential (TEP) of bronchiole. In the presence of luminal Cl^-^, the effects of both amiloride and Fsk+IBMX on TEP were almost imperceptible (left side). However, when Cl^- ^was substituted with Gluconate to more effectively reveal the Cl^- ^conductance, addition of amiloride depolarized TEP and Fsk+IBMX hyperpolarized the TEP (right side), suggesting that the large Cl^- ^conductance present in the epithelium mutes (shunts) the smaller changes in conductance occasioned by amiloride and Fsk when isotonic Cl^- ^is present bilaterally. NaGlu: Na-gluconate

When the bronchiolar lumen was perfused with NaCl Ringers, which we assumed represented insignificant ion gradients except for a small lumen (145 mM) to serosa (110 mm) Cl^- ^gradient. Despite the fact that this small Cl^- ^gradient should render the lumen positive, there was a small spontaneous lumen negative potential of about -3 mV (Fig. 2; Table 1). When we applied amiloride (10 μM) to the lumen to block Na^+ ^conductance (gNa^+^), the TEP decreased slightly, but without statistical significance (Fig. 2; Table 1). We were unable to detect a change in total conductance with amiloride applied to the lumen.

**Table 1:** Amiloride Inhibition

	NaCl	NaCl+Amil	NaGlu	NaGlu+Amil
TEP (mV)	-3.1 ± 0.6	-2.6 ± 0.7	-57.3 ± 2.7	-43.6 ± 2.7
Δ TEP (mV)	--	+0.5	--	+13.7
n	25	10	25	16
P value	--	0.676	--	<0.001

### Gluconate Substitution

**Figure 3:**
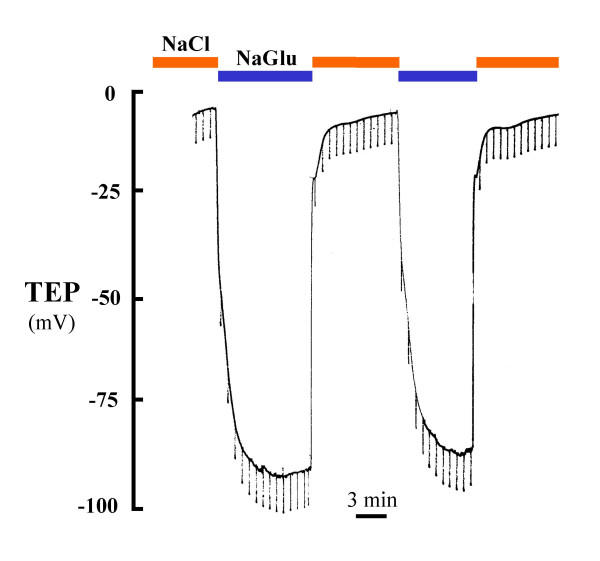
Effect of luminal Cl^- ^substitution in a perfused small bronchiole on TEP. The substitution of Gluconate, an impermeant anion, for permeable Cl^- ^in the lumen markedly hyperpolarized the TEP. This response indicates a predominant Cl^- ^conductance that appears to be constitutively active.

However, when we replaced luminal Cl^- ^with the impermeant anion, gluconate, the TEP hyperpolarized to as much as -90 mV (Fig. 3, Table 1). Addition of amiloride (10 μM) to the lumen depolarized the mean TEP of these tissues by an average of 14 mV (Fig. 2; Table 1).

### Anion Conductance Inhibitors

**Figure 4:**
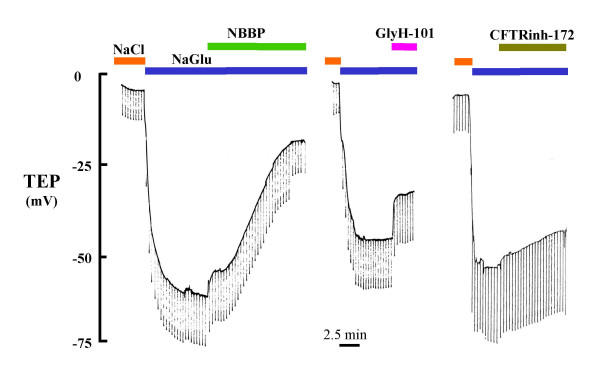
Effect of Cl^- ^conductance inhibitors on TEP. In the absence of luminal Cl^- ^(Gluconate substitution), two new inhibitors GlyH-101 (50 μM) and CFTR_Inh_-172 (5 μM, re: A. Verkman) showed at least partial inhibition of the Cl^-^:Gluconate anion diffusion potential. NPPB (100 μM) seemed to be the most effective inhibitor in terms of depolarizing the TEP.

Under conditions of symmetrical [Cl^-^] concentrations, luminal applications of anion conductance inhibitors had virtually no detectable effect on the spontaneous TEP. However, under hyperpolarizing conditions created by Cl^- ^substitution in the lumen, NPPB (100 μM), GlyH-101 (50 μM) and CFTR_Inh_-172 (5 μM) significantly depolarized the TEP by 36.7, 20.0 and 8.7 mV (Fig. 4; Table 2). Bumetanide (1 mM) had no effect on the TEP in either the presence or absence of a Cl^- ^gradient (not shown).

**Table 2:** Cl^- ^Conductance Inhibition

	CFTR_inh_-172	NPPB	GlyH-101
TEP (mV)	-48.6 ± 4.7	-16.5 ± 2.1	-26.6 ± 2.5
Δ TEP (mV)	+8.7	+36.7	+20.0
n	3	5	4
P value	0.03	0.001	0.03

### Anion selectivity

**Figure 5:**
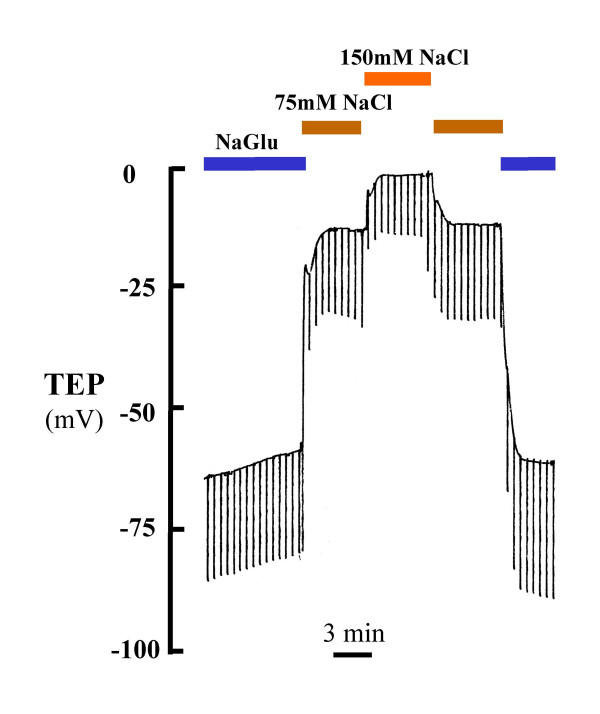
NaCl dilution diffusion across the bronchiolar epithelium on TEP. The hyperpolarization of the TEP with diluted NaCl (75 mM) indicates that Cl^- ^must be significantly more permeable through the epithelium than Na^+^.

We assayed for the relative permeability of several monovalent anions by substituting them for Cl^- ^in NaCl Ringer. Amiloride (10 μM) was added in order to block Na^+ ^transport. For luminal Cl^-^, Br^-^, I^-^, NO_3_^-^, HCO_3_^- ^and gluconate, the mean estimated P_x_/P_Cl _were, respectively, 1.0, 0.92, 0.79, 0.65, 0.33 and 0.28 (Table.3). When the NaCl Ringer perfusion solution was diluted by 1:2, the potential depolarized by 12 ± 1 mV, indicating Cl^- ^permeability exceeded the Na^+ ^permeability by about 5.4 fold (Fig. 5).

**Table 3:** Anion selectivity sequence of the perfused bronchiole. The sequence of the bronchiole (upper data) roughly fits the known sequence of CTFR in other tissues (lower data taken from literature; cf. text)

Airway Anion selectivity:	**Cl^-^**	≈	**Br^-^**	**>**	**I^-^**	**>**	**NO_3_^-^**	**>**	**HCO_3_^-^**	**>>**	**Gluconate**
Transepithelial TEP :	~0		-1.9		-5.1		-9.2		-19.5		-44.8
Estimated P_x_/P_Cl_:	1.0		0.92		0.79		0.65		0.33		0.28
											
CFTR anion selectivity:	**NO_3_^-^**	**≈**	**Br^-^**	**≈**	**Cl^-^**	**>**	**I^-^**	**>**	**HCO_3_^-^**	**>>**	**Gluconate**

Estimated P_x_/P_Cl_:	1.1		1.1		1.0		0.39		0.014		~0

### Agonists

**Figure 6:**
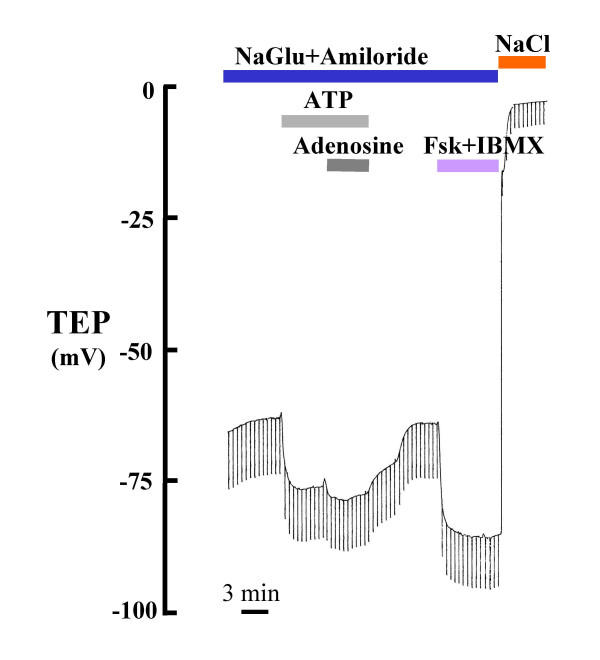
Effect of Cl^- ^conductance agonists on TEP: Applying Fsk (5 μM)+IBMX (100 μM), adenosine (100 μM), ATP (100 μM) and ATP (100 μM)+adenosine (100 μM) to the perfusate to activate CFTR gCl^- ^in the presence of a hyperpolarizing Cl^- ^gradient, the TEP hyperpolarized significantly; however, the response appeared to be increased when both ATP and adenosine were added together. In order to observe maximum effect on activation of Cl^- ^conductance, amiloride (10 μM) is present in all luminal perfusate above to block Na^+ ^conductance.

When we added forskolin (5 μM) plus IBMX (100 μM), adenosine (100 μM), ATP (100 μM) or ATP (100 μM)+adenosine (100 μM) to the perfusate to activate CFTR gCl^- ^in the presence of isotonic Cl^- ^concentrations and in the absence of a hyperpolarizing gradient, the TEP did not change perceptibly (not shown), but in the presence of the Cl^- ^gradient, the TEP hyperpolarized significantly to all agonists; the response appeared to be increased when both ATP and adenosine were added together (Fig. 6; Table 4). In order to observe the maximum effect on the activation of Cl^- ^conductance, amiloride (10 μM) was present in all luminal perfusates to block ENaC Na^+ ^conductance.

**Table 4:** Cl^-^Conductance Agonists

	Fsk+IBMX	ATP	Adenosine	ATP+Adenosine	Ionomycin
TEP (mV)	53.0 ± 2.8	-51.0 ± 0.8	-55.8 ± 6.7	-52.0 ± 6.5	-40.1 ± 7.5
Δ TEP (mV)	-26.0	-5.5	-5.5	-6.3	0.4
n	13	4	5	6	5
P value	<0.001	0.007	0.003	0.011	0.836

### RT-PCR

**Figure 7:**
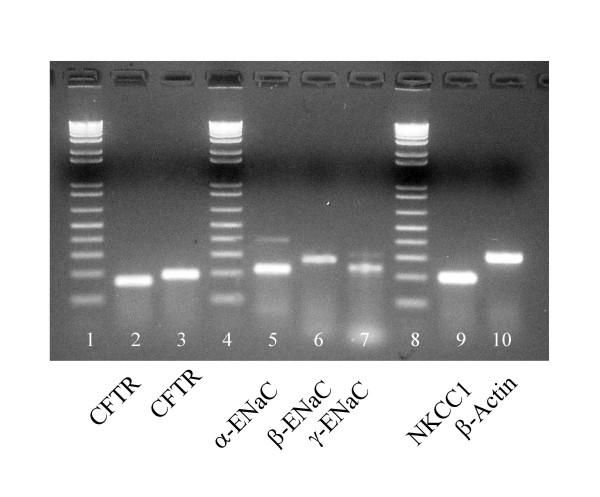
RT-PCR bands for CFTR (lanes 2 &3), α,β,γ subunits of ENaC (lanes 5,6,7 respectively), and NKCC1 (lane 9) and β-Actin (lane 10). Two independent sets of primers were used to detect CFTR. The presence of CFTR, ENaC, and NKCC1 may indicate that the epithelium has both absorptive and secretory functions. β-Actin was used as a housekeeper marker for control. Size ladders in 100 bp increments with lowest band equal to 100 bp (lanes 1,4,8).

Two sets of different primers for CFTR as well as primers for α-, β-and γ-ENaC, NKCC1, and β-Actin all produced products of predicted sizes for each of the corresponding mRNAs (Fig. 7).

### Immunocytochemistry of CFTR

**Figure 8:**
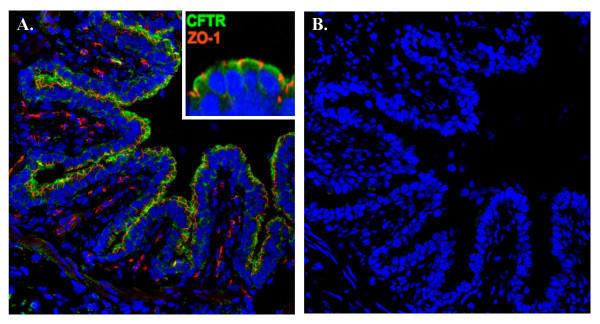
Immunocytochemical localization of CFTR in bronchiole epithelium. A.). Antibody R3194 against CFTR prominently labels (green) the apical margin of epithelial cells lining the bronchiole cut in cross section. Inset: high magnification view of epithelial cells showing demarcation of apical margin by tight junction-associated protein ZO-1 (red). B.) Control serial section stained without primary antibody.

Immunoreactive CFTR antibody (gift of C. Marino) was detected in the apical domains of the bronchiolar epithelia in a continuous border of the bronchiole. The tight junctions were labeled simultaneously and appeared as punctate areas within the border staining for CFTR consistent with the expected location for these structures (Fig. 8A and inset). Negative controls consisted of omission of primary antibodies and showed no labeling of the antibody in any tissue (Fig. 8B).

## Discussion

The airways are extraordinarily specialized conduits for air to and from the alveoli for gas exchange. They must remain moist in order to remain flexible and to effectively filter air before it enters the delicate tissues of the alveoli. Hence, a principal function of the airway epithelia is to provide and service a continuous layer of aqueous fluid on the airway surfaces. For decades, the upper airways, trachea, and large bronchi have served as models for the entire tracheobronchial epithelial function [14,22,23], and yet it is known that there are distinct regional differences progressing from nares to alveoli. For example, anatomically, the upper airways and bronchi are characterized by submucosal glands that secrete fluid directly into the airway, but are absent in the peripheral small airways [24]; [25]. Likewise, the trachea and larger airways are kept patent by cartilaginous rings whereas the peripheral airways are generally held open (but may collapse) by internal retractile forces of the lung parenchyma [26]. Similarly, the cell populations change. The epithelium of the upper respiratory tract changes from ciliated, pseudostratified columnar to simple cuboidal cells in the smaller airways where the proportion of Clara cells increase and that of ciliated cells decrease [27]. Functionally, there are differences as well. For example, in humans and in dogs, the spontaneous transmural electrical potential appears to become less negative (lumen: blood) from upper to lower airways [13,28,29]. Differences in airway surface fluid composition may also exist [30].

The functions of the lower peripheral airway epithelium is poorly defined and understood because this tissue is inaccessible and relatively unamenable to standard physiological techniques for study. Nonetheless, a few courageous attempts to unravel these mysteries have been made. About ten years ago, Ballard [13,14] and Al-Bazzaz [10] isolated and perfused small airways and bronchioles from pig and sheep, respectively. Ballard perfused porcine airways of about 1 mm diameter and reported a mean spontaneous TEP potential difference in bilateral NaCl Ringer solution of about -3.4 mV and in lumen Cl^- ^free solution of about -16 mV [14]. Al-Bazzaz perfused ovine bronchioles of about 250 μm diameter and reported a mean spontaneous TEP of -2.5 mV in bilateral NaCl Ringer and of about -4.2 mV mean TEP in lumens perfused with Cl^- ^free Ringers [11]. In all of these studies, it has been difficult to ascertain to what degree the electrical responses were muted or altered by trauma to the tissue during dissection because similar measurements are not possible *in vivo*. We, too, found similar, relatively small TEP voltages in microdissected porcine airways.

### Undissected bronchioles

Therefore, in order to maximally preserve, and minimally traumatize the airway epithelium, we avoided dissecting the bronchial structure and microperfused the airways imbedded in lung parenchyma. We found that the spontaneous TEP in bilateral Ringer solution was about -3 mV, slightly more negative than reported earlier. But in striking contrast to the previous studies (including our own dissected preparations), we found that the bi-ionic TEP with Cl^- ^free solutions in the lumen was as much as -90 mV (mean: -57 mV; Table 1; Fig. 2). These differences almost certainly reflect differences in trauma to the airway. Because of the extremely complicated morphology that arises from arborization of the airway in route to hundreds of alveolar acini, it is impossible to physically remove the surrounding tissues (very small bronchioles, respiratory bronchioles, blood vessels, and alveolar sacs) without breaking or tearing smaller "branches" from the "tree" of airways. Both Ballard and Al-Bazzaz attempted to patch these breaks by micro-suturing obviously dangling limbs; unfortunately, only larger branches are amenable to such heroic attempts and many smaller, even microscopic transepithelial openings, must remain.

In order for an epithelium to reflect its *in vivo *electrical properties *in vitro*, it is fundamental that the integrity of the epithelial sheet be conserved because TEP measurements depend on separation of charge. Breaks, holes, or tears in the barrier inescapably create electrical shunts, which, by allowing simultaneous back leak of epithelial current, prevent separation of charge. Even though the individual cells or groups of cells of the epithelia function and respond physiologically, the transepithelial voltage signals will be erroneously muted or lost through such shunts. In the present case, it appears that perfusion of the bronchiole without dissection from surrounding parenchyma, minimizes trauma induced shunts and allows detection of a much more complete and robust electrical signal. Unfortunately, the price for this preservation of signal is the inability to control the contra luminal solution. Keeping in mind that the undissected bronchiole is embedded in a mass of air filled alveoli together with numerous other elements of the tissue at this level, it is easy to see that changing the bath solution can have no acute effects on the composition of the contra luminal solution at the basilateral membrane of the epithelium. Consequently, we did not change the bath solution and assumed that normal Ringer solution is sufficiently similar to extracellular fluid *in vivo *to avoid introducing significant free solution diffusion potentials or altering the physiological composition of the native fluid present in the extracellular compartment of the in tact preparation.

### Cl^- ^Conductance

The simple fact that substituting Cl^- ^in the lumen spontaneously created a large lumen negative transmural potential (Figs. 2, 3; Table 1) immediately indicates that the small airway is characterized by a dominant constitutively active anion selective conductance. The fact that imposing a putative 1:2 NaCl diffusion gradient across the epithelium resulted in -12 mV hyperpolarization indicates that the intact bronchiole epithelium is at least 5 times more permeable to Cl^- ^than to Na^+ ^(Fig. 5). The facts that this Cl^- ^conductance is immediately evident upon initial perfusion without any prior agonist stimulation and that additions of agonists did not significantly hyperpolarize the bronchiole perfused with 150 mM NaCl and hyperpolarized bronchioles perfused with Na Gluconate only by about 20–25% demonstrates that the Cl^- ^conductance is constitutively open under these conditions (Fig. 6). That is, from the first moment of measurement after cannulation, the large Cl^- ^conductance is present (Figs. 2, 3, 4 and 5). There was no need to activate PKA or wait for the transmural potential to develop even though the addition of forskolin, adenosine, and ATP appeared to increase Cl^- ^conductance (Figs. 2, 6; Table 4). In secretory cells where secretory activity is usually an acute, temporal event, CFTR is assumed to remain closed until activated by PKA and ATP. However, in the human sweat duct, also a rich source of CFTR, where the transport function is exclusively absorptive and where CFTR is thought to be the only anion conductance through the tissue, CFTR appears with a large Cl^- ^conductance at the first moment of perfusion and measurement, indicating a constitutively open state for the CFTR channel in this tissue as well. It is well established that CFTR can be regulated in the classic sense by PKA phosphorylation and ATP in the sweat duct, but the cytoplasm must first be "rinsed" of small solutes by permeabilizing the basilateral membrane [31].

### CFTR

Since obstructive airway disease in Cystic Fibrosis arises in the small airways [32-34] and CF is known to be due to mutations in the CFTR gene that expresses a Cl^- ^channel, we asked if this conductance could be due to CFTR. We found several lines of evidence that are consistent with CFTR being responsible for the Cl^- ^conductance. First, the anion selectivity sequence, excepting NO_3_^-^, is grossly the same as that for CFTR (Table 3); i.e., Cl^- ^≈ Br^- ^> I^- ^> NO_3_^- ^> HCO_3_^- ^> Gluconate, and compares favorably with that of CFTR: SCN > NO_3_^- ^> Br^- ^> Cl^- ^> I^- ^> HCO_3_^- ^> F^- ^> ClO_4_^- ^> gluconate [35-37]. Second, it is well known that CFTR is activated characteristically by cAMP mediated protein kinase A, which is driven pharmacologically by forskolin and IBMX. Here, we see (Figs. 2, 6; Table 4) that these agonists routinely elevate the bionic Cl: gluconate diffusion potential by an average of -22 mV (n = 13). Similarly, ATP and adenosine may activate CFTR in airway epithelia since these agonists can elevate cAMP via purinergic receptors. [38-40]. In contrast, when we applied ionomycin to elevate intracellular Ca^2+^, there was no effect (not shown), suggesting that since CFTR is not sensitive to Ca^2+ ^mediated activation either, a.) the conductance is due to CFTR, b.) other Ca^2+ ^activated Cl^- ^conductances must be fully constitutively activated or not present, or c.) luminal application of the ionophore drug does not increase intracellular Ca^2+ ^effectively. Third, CFTR is known to be inhibited by NPPB, CFTR_Inh_-172, and GlyH-101[19,21]. These inhibitors had varying, but consistently inhibitory effects on the TEP (Fig. 4; Table 2) indicating inhibition of Cl^- ^conductance in the airway epithelium. However, the fact that none of the inhibitors appeared to completely inhibit the transepithelial potential might argue that another anion channel conductance is present. Two points diminish this argument. First, if the tissue is actively transporting, anion channel inhibitors will not completely ablate the TEP because that component of potential generated by the basilateral K^+ ^emf and the apical Na^+ ^emf should not be blocked and should be reflected across the epithelium in the TEP. Secondly, even when the CFTR Cl^- ^channel has been isolated from active transport components, anion channel blockers do not usually completely block its conductance in native tissue [41]. Moreover, recently, in contrast, to our results, GlyH-101 was reported to be ineffective in blocking the Cl^- ^conductance of pig nasal airways [42]. It is not known whether CFTR is present in the nasal epithelium of pigs, but biochemically, we found markers for conserved regions of expressed CFTR RNA from reverse transcriptase polymerase chain reactions (Fig. 7) in bronchioles dissected free of parenchyma post perfusion. Appropriate bands for CFTR protein in lysates of frozen peripheral lung tissue were also observed with affinity purified antibodies (J. Riordan, personal communication), and as shown in (Fig. 8) CFTR localized immunocytochemically, specifically to the apical surface of the bronchiolar epithelia. These data strongly suggest that CFTR is a part of, or probably is, the primary source of the anion conductance in small airways.

### Transport Function

Not only does the bronchiole resemble the sweat duct with respect to exhibiting a constitutively active Cl^- ^channel that responds to activation of PKA, but both are also apparently insensitive to ionomycin. They both show equally large bionic diffusion potentials for Cl^- ^and are several fold more permeable to Cl^- ^than to Na^+^, and yet both have very low transmembrane potentials in bilateral isotonic Ringer solutions [43]. Both are incompletely inhibited by anion channel blockers [20,41,44], and both are sensitive to amiloride. On the basis of these observations and by analogy with the sweat duct, it is tempting to propose that the bronchiole at least in its basal state is a constitutively absorptive epithelium.

## Conclusions

We have found that the epithelium of terminal airways of the pig appears to express an anion permeability that constitutively dominates the electroconductive properties of this zone of the airway. Its anion selectivity sequence is similar to that expected for CFTR, and its activity can be enhanced by forskolin/IBMX or decreased by anion channel blockers known to inhibit CFTR. RT-PCR amplification products and specific antibodies identify CFTR in this tissue. The small airway appears to share a number of properties with the human sweat duct and may, by analogy, belong to a class of highly absorptive epithelia.

## Abbreviations

CFTR: Cystic Fibrosis Transmembrane Conductance Regulator

ENaC: Epithelial Na^+ ^Channel

NKCC: Na^+^-K^+^-2Cl^- ^Cotransporter

TEP: Transepithelial Potential

PKA: Protein Kinase A
